# Optimization of nutrient utilization efficiency and productivity for algal cultures under light and dark cycles using genome-scale model process control

**DOI:** 10.1038/s41540-022-00260-7

**Published:** 2023-03-15

**Authors:** Chien-Ting Li, Richard Eng, Cristal Zuniga, Kai-Wen Huang, Yiqun Chen, Karsten Zengler, Michael J. Betenbaugh

**Affiliations:** 1 Department of Chemical and Biomolecular Engineering, Johns Hopkins University, 3400 North Charles Street, Baltimore, MD 21218 USA; 2 Department of Pediatrics, University of California, San Diego, 9500 Gilman Drive, La Jolla, CA 92093-0760 USA; 3 Department of Biology, San Diego State University, San Diego, USA; 4 Department of Bioengineering, University of California, San Diego, 9500 Gilman Drive, La Jolla, CA 92093-0412 USA; 5 Center for Microbiome Innovation, University of California, San Diego, 9500 Gilman Drive, La Jolla, CA 92093-0436 USA

**Keywords:** Computer modelling, Plant sciences, Metabolic engineering

## Abstract

Algal cultivations are strongly influenced by light and dark cycles. In this study, genome-scale metabolic models were applied to optimize nutrient supply during alternating light and dark cycles of *Chlorella vulgaris*. This approach lowered the glucose requirement by 75% and nitrate requirement by 23%, respectively, while maintaining high final biomass densities that were more than 80% of glucose-fed heterotrophic culture. Furthermore, by strictly controlling glucose feeding during the alternating cycles based on model-input, yields of biomass, lutein, and fatty acids per gram of glucose were more than threefold higher with cycling compared to heterotrophic cultivation. Next, the model was incorporated into open-loop and closed-loop control systems and compared with traditional fed-batch systems. Closed-loop systems which incorporated a feed-optimizing algorithm increased biomass yield on glucose more than twofold compared to standard fed-batch cultures for cycling cultures. Finally, the performance was compared to conventional proportional-integral-derivative (PID) controllers. Both simulation and experimental results exhibited superior performance for genome-scale model process control (GMPC) compared to traditional PID systems, reducing the overall measured value and setpoint error by 80% over 8 h. Overall, this approach provides researchers with the capability to enhance nutrient utilization and productivity of cell factories systematically by combining genome-scale models and controllers into an integrated platform with superior performance to conventional fed-batch and PID methodologies.

## Introduction

Microalgae represent promising microorganisms for transforming renewable resources and inorganic carbon sources into biomass, biofuel precursors, and high-value products^1^. One of the major challenges in making algal-derived products competitive against existing products in the market is developing sustainable bioprocesses that maximize productivity while also minimizing nutrient costs. Another challenging aspect of algal cultures is the presence of alternating light and dark cycles, which can result in major changes in algal physiology and metabolism^2^. Under photoautotrophic conditions, algae will grow in the light and lose biomass in the dark due to the release of carbon through respiration^3^. This can be problematic if the goal is to maximize biomass or cellular components. An alternative to this approach is to employ organic carbon sources, such as glucose, to drive biomass production during dark cycles^4^. However, most algae bioreactors operate predominantly in fed-batch mode and thus feeding can be challenging during dark cycles due to difficulties in estimating the amount of glucose organics to be added^5,6^. As a result, traditional fed batch is not an ideal control strategy to achieve efficient nutrient utilization for periodic alternating light and dark cycles due to the presence of unutilized glucose remaining in the bioreactor during the light cycles or the premature depletion of glucose before the end of the dark cycle.

In order to address this issue, we applied a genome-scale model (GSM) predictive control (MPC) strategy to optimize glucose and nitrate feeding for the model alga *Chlorella vulgaris*. In this system, during autotrophic growth, energy from light was used to capture carbon dioxide (CO_2_) to support algal growth, whereas glucose was added to supply energy to sustain algal growth during dark cycles. Over the past twenty years, constraint-based modeling has been developed and implemented to gain insights into the metabolism and the metabolic flux within different microorganisms^7^. GSMs have been constructed for multiple algal species including *Chlamydomonas*^8^, *Chlorella*^9^, and *Nannochloropsis*^10^. Recent studies have attempted to couple GSMs with dynamic flux balance analysis to optimize feeding strategies in order to improve ethanol production in *Saccharomyces cerevisiae* and *Escherichia coli* cultures in silico^11,12^. Another study used a GSM to optimize media supply and maintain *C. vulgaris* biomass^13^. The majority of these studies utilized a single model to optimize nutrient feeding, however, cells dynamically change their biomass composition under different culture conditions^14^. Previously, our group developed a genome-scale metabolic model with dynamic biomass compositions based on experimentally determined biomass measurements and corresponding omics data^15^. We then applied this dynamic model to predict nutrient requirements under autotrophic, heterotrophic, and nitrogen-limited conditions^16^.

MPC has become a widely used strategy to optimize biomanufacturing by using a model to predict cellular behavior, which is a highly nonlinear process. To utilize this approach, kinetic models have been applied with a nonlinear MPC algorithm to optimize fixation of CO_2_ in *C. vulgaris* cultures^17^. Another study applied an artificial neural network to build data-driven models and regulate light intensity for a microalgae culture^18^. Both studies demonstrated the robustness of this MPC strategy but primarily utilized kinetic equations modeling nutrient transport from extracellular environments without detailed representation of the entire metabolism. Indeed, a previous large-scale outdoor culture study indicated that the significant barrier to algal bioproduction was the lack of understanding of microalgal biology for optimal biomass generation^19^. To improve our knowledge of metabolism, GSMs have been constructed for multiple algal species by our group and others and used as tools to improve strain development, to identify metabolic bottleneck and to understand microbiome interactions, among other applications^20,21^. In this regard, GSMs have been used to predict metabolic demands under different culture conditions such as photoautrophic and heterotrophic environments, depletion of nitrogen source or for multiple species interaction^22^. This expansive description of cellular metabolism provides a valuable knowledge base that can also be used as a powerful tool for controlling cellular performance in biomanufacturing processes.

Therefore, in this study we incorporated separate photoautotrophic (*i*CZPA-T1) and heterotrophic (*i*CZH-T1) genome-scale metabolic models for light and dark cycles, respectively, in order to optimize metabolic pathways and utilization of nutrient supplies and compared their performance in terms of production of biomass, fatty acids (FAs), and lutein to standard autotrophic and heterotrophic cultivations. The metabolic models were then used to control nutrient supply in both open-loop and closed-loop configurations, which represents a useful approach to efficiently channel diverse nutrients to algal cellular components and enhance production of algal-derived bioproducts during day-night cycles. To enhance control, we adapted the parameters in the closed-loop controller based on feedback measurements of biomass and nutrients to improve model predictions for bioreactor operations. This approach of closed-loop genome model process control (GMPC) demonstrated significant improvements in nutrient utilization efficiencies compared to conventional fed-batch approaches. Finally, we demonstrated superior performance for this GMPC system compared to conventional PID systems, illustrating the value of this methodology for improving biomanufacturing processes.

## Results and discussion

### Advantages of using genome-scale model predictions on *C. vulgaris* cultures under light/dark cycles

**Fig. 1:**
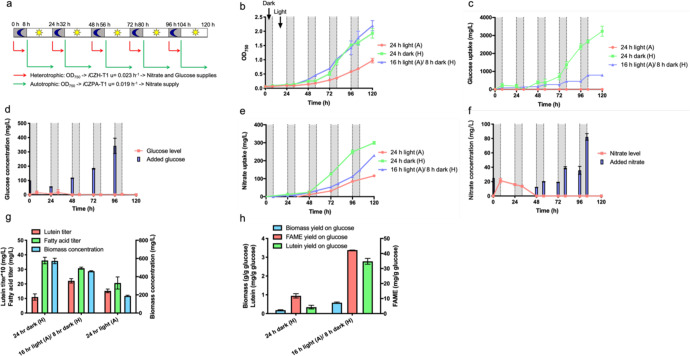
*C. vulgaris* cultures under 24 h light autotrophic conditions (24 h light (A)), 24 h dark heterotrophic conditions (24 h dark (H)) and 16 h light autotrophic/8 h dark heterotrophic cycles (16 h light (A)/8 h dark (H)) with nutrient supplies predicted by genome-scale models. **a** Flowchart of model predictions over light/dark cycles. **b** Cell growth. **c** Glucose uptake in all three conditions. **d** Glucose feed and glucose concentration in the media under 16 h light (A)/ 8 h dark (H) cycles. **e** Nitrate uptake in all three conditions. **f** Nitrate feed and nitrate concentration in the media under 16 h light (A)/8 h dark (H) cycles. **g** Biomass concentration, fatty acid titer and lutein titer. **h** Biomass yield, Fatty acid yield and lutein yield on glucose. The data represents the mean ± SD for *n* = 3 (biological triplicates).

Previously, a collection of genome-scale metabolic models (GSMs) of *C. vulgaris* has been constructed to reflect different growth phenotypes (*i*CZ946-PAT1 to PAT6 and *i*CZ946-HT1 to HT5 in Supplementary Table 1)^9,15^ and our group has successfully demonstrated the application of *i*CZ946-PAT1, HT1, and PAT5 models to optimize nutrient supply for autotrophic, heterotrophic and nitrogen limited conditions in 150 mL cultures, respectively^16^. Here, we expanded on this methodology to apply photoautotrophic (*i*CZ946-PAT1) and heterotrophic (*i*CZ946-HT1) models, in sequence to predict nutrient requirements under alternating light and dark cycles, which mimic conditions present in outdoor cultivation systems (Fig. 1a). During these cycles, glucose concentration, nitrate concentration, and biomass as OD_750_ were measured every 8 or 16 h. The OD_750_ measurements were converted to biomass concentration, which served as constraints in genome-scale metabolic models to predict nutrient requirements to support a fixed growth rate under alternating autotrophic and heterotrophic conditions. The GSM *i*CZH-T1 was applied to predict glucose and nitrate requirements during heterotrophic growth, which averaged around 0.023 h^−1^ under dark conditions and the corresponding glucose and nitrate levels were added to the cultures accordingly based on model predictions. In this way, the alternating day-night cultures received the appropriate glucose and nitrate feeds, based on the model predictions, for growth during the dark periods. Alternatively, for the light cycle, the model was switched to the photoautotrophic model *i*CZPA-T1 (see Supplementary Table 1) in order to predict nitrate feeding requirements, which averaged around 0.019 h^−1^ during the 16-hour light conditions (Fig. 1a).

The optical density following glucose and nitrate feeding based on model predictions was then compared to growth under 24-h light autotrophic (24 h light (A)) and under 24-h dark heterotrophic (24 h dark (H)) conditions without any model predictive feeding (Fig. 1b). For heterotrophic conditions, excess glucose (10 g/L) and nitrate (250 mg/L) were added to ensure no limitations during the growth period. While no glucose was present in the autotrophic cultures, the cells still grew by fixing CO_2_ and reached OD_750_ of approximately 1.0 with 250 mg/L initial nitrate added (red line in Fig. 1b, c). Alternatively, the optical density for the alternating light–dark cycling (16 h light (A)/8 h dark (H)) was comparable to that obtained by heterotrophic conditions with both reaching OD_750_ of 1.9 or higher by 120 h (Fig. 1b). Previous studies found that culturing *C. vulgaris* under heterotrophic conditions can improve its growth under subsequent autotrophic conditions^23^. Indeed, the activation of Rubisco protein under autotrophic conditions can also support higher growth rates under subsequent heterotrophic conditions^24^. These studies demonstrated the synergistic benefits from alternating light and dark cycles and helped to explain the comparable growth profiles we observed under alternating light and dark cycles (blue line in Fig. 1b) and completely dark heterotrophic conditions (green line in Fig. 1b). Alternatively, *C. vulgaris* grew more slowly under pure autotrophic conditions (red line in Fig. 1b).

For the 24 h dark (H) conditions, *C. vulgaris* consumed approximately 3200 mg/L glucose over 120 h (Fig. 1c). For the alternating light and dark cycle case (16 h light (A)/8 h dark (H)), an initial feed of 100 mg/L glucose was added to facilitate growth during the first dark cycle. Glucose levels in the media were measured before we supplemented glucose. *C. vulgaris* consumed most of the initial glucose, reducing the concentration to 16 mg/L within the first 8 h (Fig. 1d). At this point, conditions were cycled back to light and no additional glucose was added until 24 h. At 24 h, a bolus of 56 mg/L glucose was added to support heterotrophic growth based on model-predicted requirements for the next 8 h. After 32 h, the added glucose was indeed nearly consumed by the end of the second dark cycle. Glucose supplemented at the start of each dark cycle was then consumed completely over the dark cycles at 48, 72, and 96 h. Overall, the glucose consumption was reduced from 3200 mg/L for the heterotrophic cultures to 800 mg/L for the light/dark cycles within 120 h, a reduction of nearly 75% (Fig. 1c).

Nitrate consumption was also compared in the three different cultures (Fig. 1e). *C. vulgaris* consumed 120 mg/L nitrate and 300 mg/L in the autotrophic and heterotrophic cultures, respectively, over 120 h. Alternatively, for the light and dark cycling case, the basal 25 mg/L nitrate was exhausted in the media by 48 h (Fig. 1f). Subsequently, the GSMs *i*CZPA-T1 and *i*CZH-T1 were applied to estimate the nitrate required at the beginning of the light and dark periods for each cycle. This strategy resulted in no significant excess of nitrate remaining in the media at the end of both periods (Fig. 1f). As a result, around 230 mg/L nitrate was consumed by 120 h in alternating light/dark cycles, which was 23% less than the nitrate utilized in the dark heterotrophic case (Fig. 1e).

Two major microalgae-derived products of biotechnological interest, lutein, and FA, were analyzed for three different cultures in terms of total titer (mg/L in Fig. 1g) and yield per glucose (mg/g glucose in Fig. 1h). At the completion of the experiment (120 h), without light stimulation, the lutein titer was the lowest under 24 h dark (H) conditions (Fig. 1g), however, the FA and biomass concentrations were the highest at approximately 36 and 570 mg/L. The slower growth rate at the 24-h light (A) conditions resulted in the lowest FA titer and biomass concentrations. For the 16 h light (A)/8 h dark (H) conditions, FA and biomass concentrations were intermediate between the values we obtained in autotrophic and heterotrophic conditions. Interestingly, given the combinatorial effect of high biomass accumulation from heterotrophic conditions and high lutein content from photoautotrophic light cycles, the highest lutein titer of 2.2 mg/L was achieved under alternating light and dark cycles (Fig. 1g). Previous studies indeed found that light is an important factor to stimulate lutein production in microalgae^25^. Overall lutein, FA and biomass yields per gram of consumed glucose increased significantly between three and eightfold under alternating light and dark cycles as compared to heterotrophic conditions (Fig. 1h). During the light and dark cycling, *C. vulgaris* only needed the glucose supply for 8 h of the dark period every 24 h, which led to more efficient glucose utilization.

In this way, our results showed that total biomass, lutein, and FA titers were all higher for light–dark cycling compared with autotrophic cultures (Fig. 1g) and product yields per gram of glucose were all higher for light cycling than heterotrophic cultures (Fig. 1h). Previous omic^26^ and fermentation studies^27^ typically compared mixotrophic with autotrophic or heterotrophic conditions because they did not have the capability to control nutrient supply accurately under alternating light and dark cycles. However, both metabolic pathways are important for *C. vulgaris*. Autotrophic metabolism provides algae with the capability to fix carbon dioxide and synthesize high amount of carotenoids such as lutein in order to protect the free radicals generated during photosynthesis. Heterotrophic metabolism provides algae with the capacity to utilize organic carbon for efficient biomass and FA generation. By taking advantages of both metabolic capacities, our nutrient control approach clearly exemplifies the benefits of genome-scale metabolic models to optimize nutrient supply for maximizing biomass and product yields under light and dark cycles.

### Comparing the open-loop genome-scale model process control with fed-batch control on *C. vulgaris* cultures

**Fig. 2:**
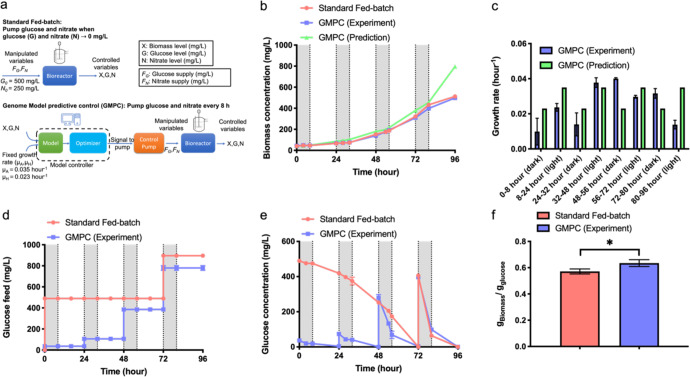
Comparison between open-loop genome-scale model process control (GMPC) with standard fed-batch cultures. **a** Flowchart of standard fed-batch and open-loop GMPC cultures. **b** Cell growth. **c** Growth rate comparison between GMPC (Experiment) and GMPC (Prediction). **d** Glucose supply during the cultures. **e** Glucose level in the media. **f** Biomass yield on glucose. The data represents the mean ± SD for *n* = 3 (technical triplicates). *P ≤ 0.05 (GraphPad unpaired *t* test).

After demonstrating the capability of using these genome-scale metabolic models to control nutrient supply against autotrophic and heterotrophic systems, we next compared the performance of cultures using genome-scale model process control (GMPC) with traditional fed-batch cultures for alternating photoautotrophic light and heterotrophic dark cycles. The experiment was performed in a 2 L bioreactor system with computer-controlled glucose and nitrate supply using the Matlab^TM^ software (MathWorks) to run GSMs and control two peristaltic pumps. The standard (non-model controlled) fed-batch bioreactor was initially supplied with 500 mg/L glucose with additional glucose supplied to the bioreactor periodically in 500 mg/L bolus increments as the glucose was exhausted (Fig. 2a). Alternatively, an open-loop model control system relied on Matlab-based GSM predictions to control the nutrients required for growth without any inputs of measurements from the system. This algorithm runs by setting the initial biomass concentration (X_0_), initial nitrate concentration (N_0_), and glucose concentration (G_0_), which are three controlled variables in the system, along with fixed photoautotrophic and heterotrophic growth rates, which were based on previous test runs. The computer then runs the GSM to predict the nitrate and glucose requirements, which dictated manipulated variables (*F*_G_, *F*_N_) to be supplied automatically every 8 and 16 h for a period of 4 days.

*C. vulgaris* grew similarly in both standard fed-batch culture and GMPC culture with the biomass concentration reaching around 500 mg/L at 96 h (Fig. 2b). However, a significant deviation was observed between the GMPC prediction (green) and experimental results (blue) after 80 hours of culture. Measured growth of *C. vulgaris* was significantly lower than model simulation, indicating an inconsistency between model predictions and experimental results during this time period. A growth rate comparison between GMPC prediction and experimental results for individual growth periods, including heterotrophic and autotrophic cycles, is shown in Fig. 2c. The experimental growth rate of autotrophic cultures (blue bars in Fig. 2c) gradually declined from 0.038 h^−1^ at 32–48 h to 0.030 h^−1^ at 56–72 h and eventually to 0.014 h^−1^ at 80–96 h, which was 60% lower than the model prediction of approximately 0.035 h^−1^ (green bars in Fig. 2c). Previous studies have observed that progressive increase in biomass will block light penetration and thus alter algal growth^28^, which may explain the gradual decline in the algal growth rate and resulting deviations away from model predictions, as this effect was not considered in our open-loop model predictive control. Furthermore, the heterotrophic growth rate was around 0.04 h^−1^ at 48–56 h and 0.032 h^−1^ at 72–80 h, which, in contrast, was higher than the model prediction of around 0.023 h^−1^ at these time points (Fig. 2c). Previous investigations have indicated that CO_2_ fixation is still active up to 18 h after transferring from light-facilitated photoautotrophic to heterotrophic conditions^24^, which may explain the higher growth rate observed in our measurements compared to the predictions under purely heterotrophic conditions.

For the GMPC case, 36 mg/L glucose was supplied initially based on model predictions of the amount to be consumed over the 8 h heterotrophic growth followed by additional supplements at 24, 48, and 72 h (Fig. 2d). However, glucose was not consumed completely in GMPC cultures at 32, 56, and 80 h with 40, 70, and 100 mg/L glucose, respectively, was left in the media, indicating limitations for these open-loop model predictions (Fig. 2e). In the standard fed-batch cultures, a bolus of 500 mg/L glucose was fed to the standard fed-batch culture at 0 h and completely utilized by *C. vulgaris* at around 72 h, followed by the addition of another 500 mg/L glucose supplement at that time (Fig. 2d). As a result, *C. vulgaris* was likely consuming some glucose and CO_2_ simultaneously during the light cycle, resulting in mixotrophic conditions for both the GMPC case and standard fed-batch cultures. Indeed, we observed declines in the glucose levels of *C. vulgaris* during the light cycles in both cases (white sections in Fig. 2e). This may explain the similar cell growth curves of *C. vulgaris* between standard fed-batch cultures and GMPC cultures (Fig. 2b). However, even though *C. vulgaris* consumed glucose in both conditions, the biomass yield per gram of glucose was still 12% higher for the GMPC group (0.63 g_Biomass_/g_glucose_) compared to the fed-batch group (0.57 g_Biomass_/g_glucose_) (*p* < 0.05) (Fig. 2f).

The extra glucose measured (Fig. 2e) and the variation in growth predictions (Fig. 2b) suggest that there are deficiencies in the model’s capacity to predict growth and glucose consumption in this open-loop control system. In addition, the performance of open-loop GMPC in terms of biomass yield on glucose was only moderately better than fed-batch cultures, likely in part because the glucose supply was not controlled appropriately under dark cycles for the GMPC conditions. Consequently, a closed-loop feedback control loop, which was adapted to compensate for limitations in the model’s ability to describe growth at higher algal densities and mixotrophic conditions was implemented next as an improved GMPC framework.

### Closed-loop GMPC achieved better performance than standard fed-batch control

**Fig. 3:**
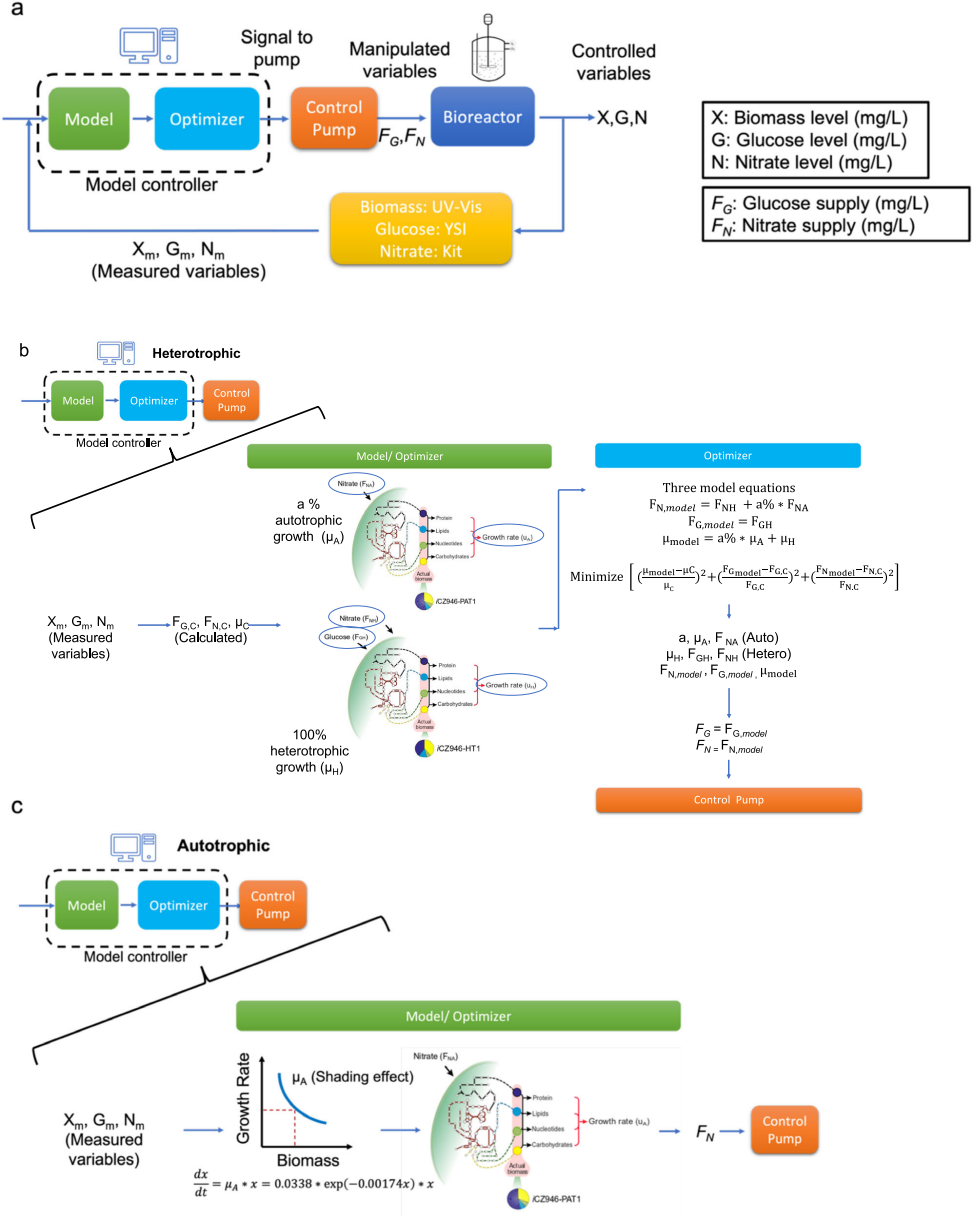
Closed-loop GMPC. **a** Flowchart. **b** Model controller in heterotrophic dark cycles. **c** Model controller in autotrophic light cycles.

To further improve GMPC, we incorporated measured data to build a closed-loop model prediction algorithm that incorporates biomass (X_m_), nitrate (N_m_), and glucose (G_m_) levels measured every 8 h (Fig. 3a). The measurements were then used as inputs into the model (green box in Fig. 3a). Unlike the open-loop system in Fig. 2, we considered CO_2_ fixation by active Rubisco protein in heterotrophic cycles, which was previously observed for algal two-stage cultivations (photoautotrophic phase-heterotrophic phase)^24^. For the heterotrophic cycles, the calculated growth rate (μ_C_), calculated glucose demand (*F*_G,C_), and calculated nitrate demand on a per L basis (*F*_N,C_) during the 8-h period were determined based on measured inputs (X_m_, G_m_, N_m_). Next, the model and algorithm optimizer (green and blue boxes in Fig. 3b) then estimate the fractions of photoautotrophic growth and heterotrophic growth in order to determine the feed under heterotrophic (dark) conditions based on the algorithm shown in Fig. 3b. The algal cells were assumed to operate under two different types of metabolism in the simulations for the dark cycle. One fraction of algal cells was assumed to grow strictly heterotrophically, as represented by model *i*CZH-T1. In addition, a certain fraction (*a*) of algal biomass was assumed to grow mixotrophically and thus fixes CO_2_ during the dark cycle as suggested in previous publications^24^. The current photoautotrophic genome-scale model *i*CZPA-T1 was not constructed to function when the light intensity (PRISM_solar_litho) is set as zero in the model. In this simulation, we therefore set the light intensity in the *i*CZPA-T1 model to a minimum for the current simulations in dark periods of the cycle. As a result, three equations were added to consider this combined metabolic operation and its impact on growth rate, glucose consumption rate, and nitrate consumption rate. The algorithm optimizes six variables including autotrophic growth rate (μ_A_), autotrophic nitrate demand (*F*_NA_), autotrophic biomass percentage (a), heterotrophic growth rate (μ_H_), heterotrophic glucose demand (*F*_HG_), and heterotrophic nitrate demand (*F*_NG_) to minimize the difference between model simulations and experimental growth rate as well as glucose and nitrate demand for the most recent 8-h dark cycle. Those optimized parameters were then used to dictate overall glucose demand (*F*_G_) and nitrate demand on a per L basis (*F*_N_) in the next 8 h, which were then controlled by the pumps. Based on the predictions, the control pump supplies glucose and nitrate to the bioreactor.

Alternatively, in the photoautotrophic phase, a differential equation for cell mass accumulation with respect to time was incorporated, which includes a term to describe the logarithmic decay of cell growth rate that occurs at increasing biomass concentrations due to light shading (Fig. 3c)^28^. This equation was built based on our experimental biomass measurements from a separate autotrophic culture run. The calculated growth rate was then used in *i*CZPA-T1 to predict and optimize nitrate supply during the light cycles.

**Fig. 4:**
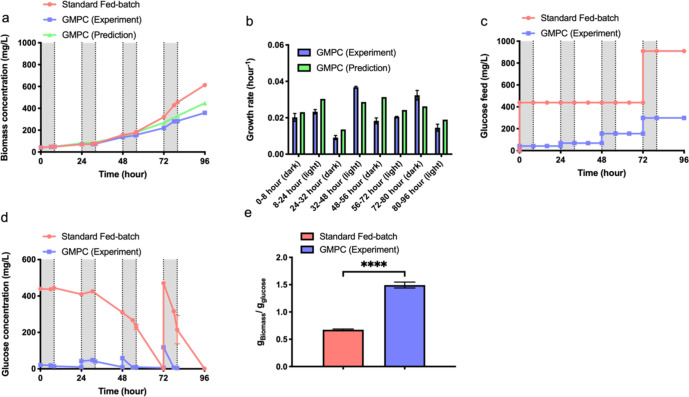
Comparison between closed-loop GMPC with standard fed-batch cultures. **a** Cell growth. **b** Growth rate comparison between GMPC (Experiment) and GMPC (Prediction). **c** Glucose supply during the cultures. **d** Glucose level in the media. **e** Biomass yield on glucose. The data represents the mean ± SD for *n* = 3 (technical triplicates). ****P ≤ 0.0001 (GraphPad unpaired t test).

The GMPC culture was then compared with a standard fed-batch culture similar to the conditions used in the open-loop experiment (Fig. 4a). Unlike the open-loop system, algal growth for the GMPC culture (blue line in Fig. 4a) was slower than the standard fed-batch culture (red line in Fig. 4a) after 56 h and eventually reached 360 mg/L for GMPC conditions versus 610 mg/L for fed-batch cultures at 96 h due to the greater stringency of glucose control during dark periods. Furthermore, the growth prediction for the closed-loop system (green line) was more closely aligned to the experimental growth rate for the GMPC cultures after 80 h (Fig. 4a) compared with the growth prediction (green line in Fig. 2b) and experimental findings for the open-loop system (blue line in Fig. 2b). Previous studies indicated the success of model predictive control is contingent on a robust process model and on-line measurements^29,30^. Indeed, in our closed-loop system, the model predictive algorithm was modified based on experimental measurements of cell density, glucose, and nitrate for both autotrophic and heterotrophic conditions in order to predict nutrient requirements for every cycle for the closed-loop system.

The growth rates between simulation and experimental results were compared for individual time periods of the cycling photoautotrophic and heterotrophic cultures (Fig. 4b). Both the model predictions and experimental growth rates changed dynamically over different heterotrophic and autotrophic cycles. The model predictions (green bars in Fig. 4b) for the growth rate during the dark cycles varied between 0.014 h^−1^ (24–32 h) and 0.031 hour^−1^ (48–56 h) over the course of the 96 hours experiment. For the experiment, the growth rates (blue bars in Fig. 4b) also changed across the different time periods with a max around 0.032 h^−1^ and a min around 0.009 h^−1^. Meanwhile, the model predictions for growth during the light cycles gradually declined from 0.030 h^−1^ at 8–24 h and eventually falling to 0.019 h^−1^ at 80–96 h. The experimental growth rates followed the same trend, decreasing from 0.037 h^−1^ at 32–48 h to 0.015 h^−1^ at 80–96 h, which again reflects the light shading effect on algal cultures.

Importantly, a total of 910 mg/L glucose was fed to the standard fed-batch as compared to 300 mg/L glucose for GMPC cultures by 96 h (Fig. 4c), a 3-fold reduction in the total glucose consumed. Furthermore, most of the glucose fed to GMPC cultures was consumed almost completely during the dark cycles, with a minimum of 6 mg/L remaining at 78 h and a maximum of 40 mg/L left at 32 h in GMPC cultures (Fig. 4d). For the fed-batch cultures, 440 mg/L glucose was consumed completely at 72 h, leading to the need for a second glucose feed around 500 mg/L. Due to the efficient glucose utilization occurring during the dark cycles of this closed-loop control system, the biomass yield on glucose increased dramatically by 2.2-fold (122%) from 0.67 g/g in standard fed-batch cultures to 1.49 g/g in the closed-loop GMPC system (Fig. 4e). In contrast, the open-loop GMPC system only had a modest 10.6% increase in biomass yield on glucose for the open-loop system (Fig. 2f).

Overall, the closed-loop GMPC demonstrated more accurate controller performance than the open-loop GMPC system. This closed-loop GMPC system relies on glucose and nitrate off-line measurement and those two measurements can take around 30 min. To address this technical challenge, other more rapid nutrient and metabolite measurement tools could be integrated such as in situ Raman spectrometry for metabolite measurements^31,32^. Alternatively, off gas analysis can be used to characterize cell metabolism toward biomass accumulation or lipid synthesis^33^ for future versions of GSM control.

### GMPC is highly robust in comparison to a PID control during dark heterotrophic periods

**Fig. 5:**
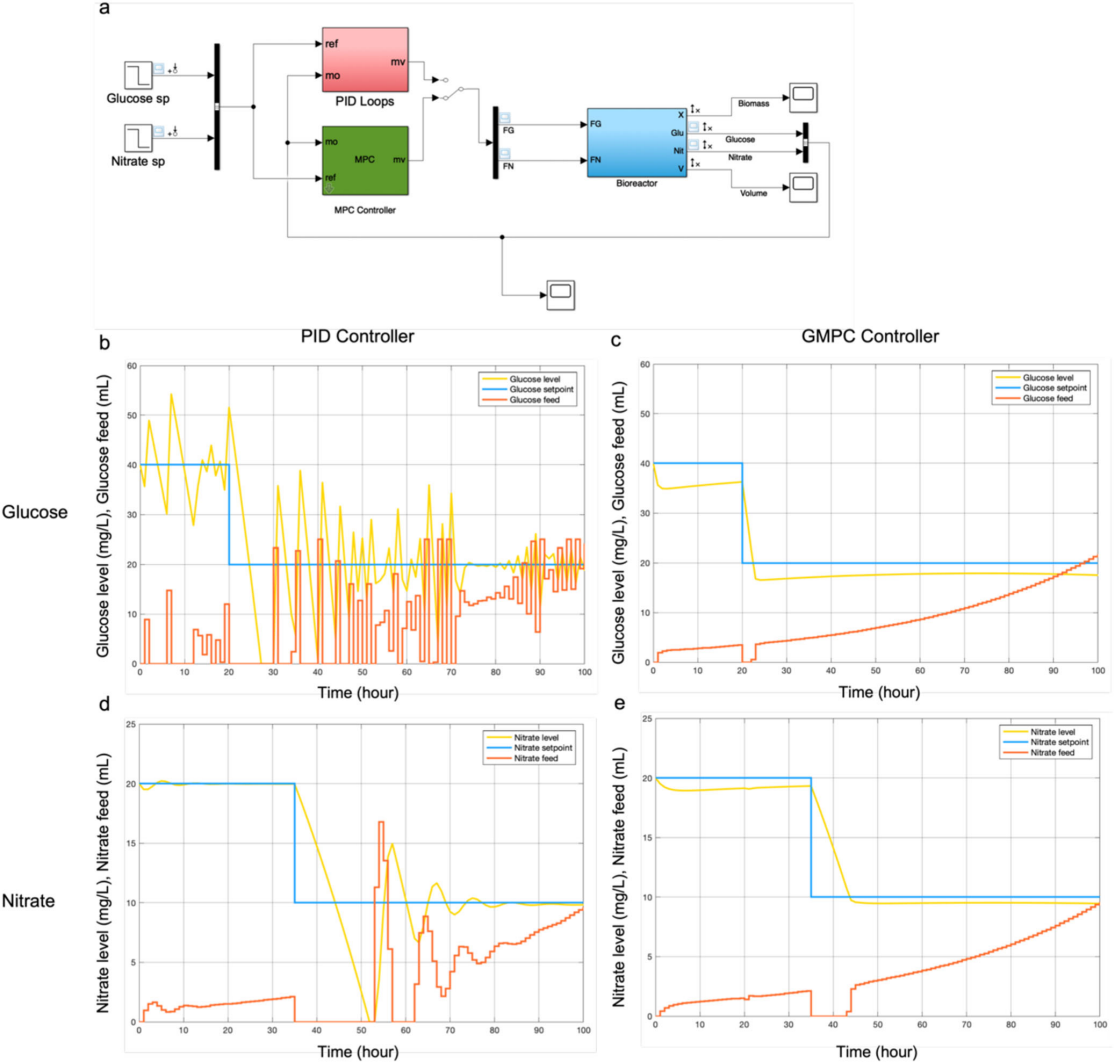
Simulation of different process control algorithms. **a** Simulink model for model predictive control and PID control. Glucose control: **b** PID controller. **c** GMPC controller. Nitrate control: **d** PID controller. **e** GMPC controller.

After demonstrating the advantages of closed-loop model prediction and its associated higher efficiency of biomass productivity with respect to glucose fed, the model predictive controller was compared to a standard PID controller in silico and experimentally. Using Simulink^TM^, a kinetic model consisting of four ODE equations was incorporated in order to describe changes in biomass, nutrient levels, and medium volume during the heterotrophic dark periods in a bioreactor (Supplementary Fig. 2). Genome-scale metabolic models were then used to determine the relationship between growth rate, glucose uptake rate, and nitrate uptake rate as described previously^16^. Next a PID controller and an GMPC controller were used to control glucose and nitrate levels separately in the bioreactor (Fig. 5a and Supplementary Fig. 2). In order to simulate the performance of the PID and GMPC controllers, a setpoint change was introduced to glucose and nitrate control around 20 and 35 h, respectively (Fig. 5b–d). The glucose setpoint was decreased from 40 to 20 mg/L (blue lines in Fig. 5b, c) and the nitrate setpoint was decreased from 20 to 10 mg/L (blue lines in Fig. 5d, e). Both PID and GMPC controllers were simulated to control glucose supply and nitrate supply every hour. The simulated glucose and nitrate levels exhibited damped oscillations when using a PID controller, a common response for this controller type (yellow lines in Fig. 5b, d). In contrast, employing the GMPC controller eliminated the damping issues and enabled the glucose and nitrate level to more rapidly reach values near the target levels (yellow lines in Fig. 5c, e). In the PID controller, the damping amplitude of glucose level was around 0–20 mg/L (Fig. 5b). In comparison, in the GMPC controller, the error in the glucose levels was around 0–5 mg/L (Fig. 5c). In the same vein, the nitrate level also exhibited damping oscillations around 0–20 mg/L after the setpoint changed to 10 mg/L at 35 h in the PID control system (Fig. 5d) while the error in the GMPC controller was less than 1 mg/L after 44 h (Fig. 5e). The total error between the setpoint and the value was around 50% and 80% lower for the glucose and nitrate levels in the GMPC simulations compared to PID controller systems following the setpoint change. Meanwhile, the glucose supply and nitrate supply increased gradually in the GMPC controller (red lines in Fig. 5c, e), while the nutrient supplied oscillated widely in the PID controller because the PID controller could not predict nutrient requirements. Instead, the amount added (red lines in Fig. 5b, d) was based on the error and the three different gains in the controller.

**Fig. 6:**
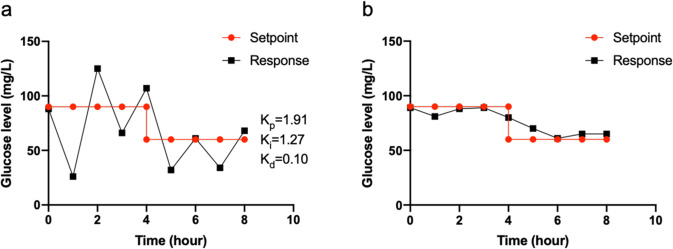
Glucose level in the bioreactor. **a** PID controller. **b** GMPC controller.

Step changes in the set point of the glucose were also tested experimentally in 2 L bioreactors to compare the effectiveness of both PID control and closed-loop GMPC on glucose concentration under heterotrophic dark conditions as a test case. The initial biomass was around 400 mg/L and nitrate was held at substantial levels (data not shown), such that nitrate concentration was not a limiting factor in this experiment. The glucose setpoint was changed from 90 to 60 mg/L at 4 h (Fig. 6). The PID controller gains were tuned on Matlab^TM^ to achieve optimal performance with proportional gain (*K*_p_), integral gain (*K*_i_) and derivative gain (*K*_d_) equal to 1.91, 1.27, and 0.10, respectively (Fig. 6a). The glucose levels were measured every hour and the data was fed to both the PID controller and the closed-loop GMPC controller with a pure heterotrophic model since light and dark cycles were not presented. The feedback signal could compensate for the modeling errors and also help to reject the disturbance in the GMPC controller. For the PID controller, the glucose levels exhibited damping oscillations around 90 mg/L from 0 to 4 h with amplitude of the oscillations around 30–60 mg/L, which was 33–66% of the setpoint level (Fig. 6a). After the setpoint changed to 60 mg/L at 4 h, the glucose level was still oscillating around the setpoint. However, for the closed-loop GMPC controller, the glucose level was controlled around 90 mg/L from 0 to 4 h with the largest error around 10 mg/L at 4 h (11% of setpoint in Fig. 6b). After the setpoint change, the glucose level gradually decreased and was stably controlled. The overall error between glucose measured value and setpoint between 0 and 8 h was 203 mg/L for PID and 43 mg/L for closed-loop GMPC, a decrease of nearly 80% for the model based controller approach.

Overall, both Simulink^TM^ simulations and experimental results demonstrated that the GMPC approach provided more robust and precise control than traditional PID controllers. While the model could anticipate the future behavior of the fermentation and take appropriate control action, the PID controller did not have this capability resulting in oscillations and overshoot behavior in both simulations and experiments. Thus, our study demonstrates how GMPC systems can serve as a bridge between genome-scale metabolic modeling and control algorithms. Since the cultivation conditions can change and affect algal cellular metabolism, our system connected feedback measurements with genome-scale metabolic models and achieved more efficient nutrient utilization and higher product yields for dynamic algal cultivation conditions. In this way, genome-scale metabolic models can be effectively utilized to improve biomanufacturing of microalgae and other industrially important microbial cell factories.

## Conclusions

Fed-batch cultivation and PID controllers have been widely used in bioprocess development. Unfortunately, fed-batch cultivation often results in poor nutrient control and wasted nutrients and conventional PID control can lead to oscillating cell behaviors and poor performance under dynamic conditions. In this study, we have utilized the power of genome-scale metabolic models to predict and control glucose and nitrate supply for *C. vulgaris* cultures under light and dark cycles and compared this approach to conventional autotrophic and heterotrophic processes. Our results first showed that utilizing genome scale models to track and limit glucose and nitrate feeding led to higher titers of biomass, FAs, and lutein than autotrophic conditions and more efficient glucose utilization and higher product yields than heterotrophic conditions. Next, implementing these models into an open loop system modestly improved performance. However, implementing a complete closed-loop system that incorporates glucose and nitrate feed optimizer for dark cycles and accounts for shading during light cycles increased biomass yield 122% more than normal fed-batch cultures. Finally, both computational simulations and experimental results demonstrated that this genome-based MPC system exhibits superior controller performance compared to conventional PID methods.

## Methods

### Algal strain and cultivation conditions

Green microalgae *C. vulgaris* UTEX 395 was obtained from the Culture Collection of Algae at the University of Texas at Austin and maintained on sterile agar plates (1.5% w-v) containing Bold’s Basal Medium (BBM). Liquid cultures were inoculated with a single colony in 12.5 mL of sterile BBM. Cells were transferred to 150 mL cultures or 2 L bioreactors at 25 °C using BBM. The growth of the cultures was monitored by measuring optical density (OD) at 750 nm. All the 150 mL cultures were done in biological triplicates at a flow rate of 13 mL/min and 2 L bioreactor cultures were done in technical triplicates at a flow rate of 400 mL/min in this study. Autotrophic cultures were grown with 5% CO_2_ under 30 μE m^−2^ s^−1^ fluorescence illumination for 125 mL cultures and 100 μE m^−2^ s^−1^ fluorescence illumination for 2 L cultures Heterotrophic cultures were grown with ambient air (0.04% CO_2_) under complete dark conditions. For alternating light and dark cycles, autotrophic conditions were used for light sections and heterotrophic conditions were used for dark sections.

### Measurement of biomass, glucose, nitrate, FA and lutein

Liquid cultures were harvested using a high-speed centrifuge (Beckman J2–21, Baltimore, USA) at 4000 × *g* for 10 min. The pellets were stored at −80 °C and lyophilized for 24 h at −40 °C under freeze-dried machine. The lyophilized algal dry biomass was weighted gravimetrically using an analytical balance. Cell culture supernatant samples were stored in 4 °C fridge for glucose and nitrate analysis. The glucose concentration was measured using YSI 2700 biochemistry analyzer (Yellow Springs, OH). The nitrate concentration was measured using nitrite/nitrate Colorimetric Assay Kit (Cayman 780001).

FAME production followed the procedure provided by Dong et al.^34^. The dry biomass samples (6–7 mg) were mixed with 0.1 mL heptadecanoic acid (5 mg/mL in methanol) (internal standard), 1 mL of chloroform/ methanol and 1.5 mL of HCl/ MeOH (5% v/v). The mixture was heated at 85 °C for 1 h. Then, the mixture was mixed with 1 mL hexane and 5 mL saturated NaCl. The solution was centrifuged at 6000 rpm for 10 min to collect hexane phase. Then, FAMEs were analyzed using an Agilent’s gas chromatography (GC) system with discharge ionization detection equipped with a capillary column (Stabilwax-DA, 30 m 0.25 mm ID, film thickness 0.25 mm). GC inlet was set at 250 °C and the injections were in a volume of 1 μL. The temperature program started at 50 °C and then increased to 170 °C at a rate of 20 °C min^−1^, with a plateau for 1 min. After this plateau, the temperature increased from 170 to 220 °C at a rate of 4 °C min^−1^ and then kept constant for 14 min. The total analysis time was 35 min. Helium was used as carrier gas.

Lutein extraction followed the procedure provided by Yuan et al.^35^. The dried algae pellets (5–10 mg) were homogenized using a mortar and pestle with 4 mL extraction solvent, the mixture of dichloromethane (25%) and methanol (75%), for 2 min and 2 times. The extraction solution was centrifuged at 10,000×*g* for 10 min and kept in dark in −20 °C. The solution was filtered before HPLC analysis. The mobile phases are eluent A (dichloromethane: methanol: acetonitrile: water, 5.0:85.0:5.5:4.5, v/v) and eluent B (dichloromethane: methanol: acetonitrile: water, 25.0:28.0:42.5:4.5, v/v).

### Genome-scale metabolic model prediction and bioreactor setup

For small-scale 150 mL cultures, biomass concentrations were input into the model to predict nutrient supplies and added to cell cultures manually. For 2 L bioreactor cultures, open-loop and closed-loop algorithms were written in Matlab^TM^ language.

The *i*CZ946 model, including six different biomass compositions for autotrophic conditions (PAT1-PAT6) and five different biomass compositions for heterotrophic conditions (HT1-HT5), was obtained from Zuniga et al.^15^ (Supplementary Table 1). GSM simulations were performed using the Gurobi Optimizer Version 5.6.3 (Gurobi Optimization Inc., Houston, Texas) solver in Matlab^TM^ (The MathWorks Inc., Natick, MA) with the COBRA Toolbox.

The experimental setup is shown in Supplementary Fig. 1. Biomass concentration (X), glucose level (G), and nitrate level (N) were chosen as controlled variables in the culture and those data were collected every 8–16 h manually. The manipulated variables were glucose demand (*F*_G_) and nitrate demand on a per L basis (*F*_N_) for 8-h period. Two pumps were used to control both variables automatically by Matlab^TM^ through Arduino chip. All the control algorithms were run on Matlab^TM^ and the codes are provided in Supplementary information.

The Simulink^TM^ simulation is shown in Fig. 5a and Supplementary Fig. 2. The blue box in Fig. 5a describes the bioreactor behavior. Four equations were built inside the blue box as shown in Supplementary Fig. 2. The inputs were *F*_G_ and *F*_N_. The outputs were biomass, nitrate level, glucose level and volume. Only nitrate levels and glucose levels were fed into the PID and GMPC controller. For the proportional-integral-derivative (PID) controller, the proportional gain (*K*_p_), integral gain (*K*_i_) and derivative gain (*K*_d_) equal to 1.91, 1.27, and 0.1, respectively. The PID controller and GMPC controller were used to control glucose supply and nitrate supply every hour in both simulation and experiment. Changes in the setpoint for glucose were introduced to see how both PID and GMPC responded to those changes.

### Summary of equations for 2L bioreactor cultures

#### Open-loop system

Initial biomass levels (*x*_0_), glucose levels (*G*_0_) and nitrate levels (*N*_0_) were measured as described above and used as inputs into the open-loop system.

$${{{\mathrm{Initial}}}}\;{{{\mathrm{conditions}}}}\!:x_{\rm{initial}} = x_0G_{\rm{initial}} = G_0N_{\rm{initial}} = N_0$$

Three equations shown below were used to predict biomass growth, nitrate consumption rate, and glucose consumption rate in the open-loop system. The growth rates under light and dark cycles were determined based on previous experimental data. After that, the growth rates were constrained in the autotrophic and heterotrophic GSMs, respectively to determine nutrient exchange rates (*r*_N_ and *r*_G_) under light and dark cycles. The methods for using growth rate to estimate nutrient exchange rates have been described previously in Chen et al.^36^. We assumed a rapid switch to a new operational steady state following the transition between light and dark cycles.

$$\begin{array}{l}\frac{{dx}}{{dt}} = \mu x\quad \quad {\rm{Light}}\;{\rm{cycle}}\;\mu = 0.035\left( {\rm{h}}^{-1} \right)\\ \quad \quad \quad \quad \quad \;{\rm{Dark}}\;{\rm{cycle}}\;\mu = 0.023({\rm{h}}^{-1})\\ \quad \quad \quad \quad \quad \;{\rm{Biomass}}\;x\left( {\frac{{gDw}}{L}} \right)\end{array}$$

$$\begin{array}{l}\frac{{dN}}{{dt}} = r_{\rm{N}}x\quad {\rm{Light}}\;{\rm{cycle}}\;r_{\rm{N}} = 0.1823\left( {\frac{\rm{mmol}}{{gDw \ast {\rm{h}}}}} \right)\\ \quad \quad \quad \quad \quad {\rm{Dark}}\;{\rm{cycle}}\;r_N = 0.1417\left( {\frac{\rm{mmol}}{{gDw \ast {\rm{h}}}}} \right)\\ \quad \quad \quad \quad \quad {\rm{Nitrate}}\;{\rm{level}}\;{\rm{N}}\left( {\frac{\rm{mmol}}{L}} \right)\end{array}$$

$$\begin{array}{l}\frac{{dG}}{{dt}} = r_{\rm{G}}x\quad {\rm{Light}}\;{\rm{cycle}}\;r_{\rm{G}} = 0\left( {\frac{\rm{mmol}}{{gDw \ast {\rm{h}}}}} \right)\\ \quad \quad \quad \quad \quad {\rm{Dark}}\;{\rm{cycle}}\;r_{\rm{G}} = 0.6426\left( {\frac{\rm{mmol}}{{gDw \ast {\rm{h}}}}} \right)\\ \quad \quad \quad \quad \quad {\rm{Glucose}}\;{\rm{level}}\;{\rm{G}}\left( {\frac{\rm{mmol}}{L}} \right)\end{array}$$

Based on the results, two pumps will pump glucose and nitrate every 8 h during the day and night cycles. The bolus cellular nitrate demand (*F*_N_) and the cellular glucose demand (*F*_G_) in mg/liter fed were determined based on the following two equations.

$$\begin{array}{l}F_{\rm{N}} = Mw_{{\rm{nitrate}}}{\displaystyle{\int}}_t^{t + 8} {\frac{{dN}}{{dt}}dt = Mw_{\rm{nitrate}}{\displaystyle{\int}}_t^{t + 8} {r_{\rm{N}}xdt} } \\ F_{\rm{G}} = Mw_{\rm{glucose}}{\displaystyle{\int}}_t^{t + 8} {\frac{{dG}}{{dt}}dt = Mw_{\rm{glucose}}{\displaystyle{\int}}_t^{t + 8} {r_{\rm{G}}xdt} } \\ F_{\rm{N}}\!\!:{\rm{Nitrate}}\;{\rm{demand}}\;{\rm{every}}\;8\;h\;{\rm{(mg/L)}}\\ F_{\rm{G}}\!\!:{\rm{Glucose}}\;{\rm{demand}}\;{\rm{every}}\;8\;{\rm{h}}\;{\rm{(mg/L)}}\end{array}$$

The peristaltic pumps will pump nitrate and glucose into the bioreactor (*T*_pump,N_, *T*_pump,G_) based on nitrate and glucose demand on a per liter basis (*F*_N_, *F*_G_ (mg/L)), feed stock concentration (*C*_N_, *C*_G_ (mg/mL)), volume of the bioreactor (*V* (*L*)) and pump volumetric flow rate speed (*Q* (mL/s)).

$$\begin{array}{l}T_{\rm{pump}},_{\rm{N}} = \frac{{F_{\rm{N}}\left( {\frac{\rm{mg}}{\rm{L}}} \right) \ast V(L)}}{{C_{\rm{N}}\left( {\frac{\rm{mg}}{\rm{mL}}} \right) \ast Q\left( {\frac{\rm{mL}}{\rm{s}}} \right)}} = \frac{{F_{\rm{N}}\left( {\frac{\rm{mg}}{\rm{L}}} \right) \ast 2(L)}}{{5\left( {\frac{\rm{mg}}{\rm{mL}}} \right) \ast 3\left( {\frac{\rm{mL}}{\rm{s}}} \right)}}\\ T_{\rm{pump}},_{\rm{G}} = \frac{{F_{\rm{G}}\left( {\frac{\rm{mg}}{\rm{L}}} \right) \ast V(L)}}{{C_{\rm{G}}\left( {\frac{\rm{mg}}{\rm{mL}}} \right) \ast Q\left( {\frac{\rm{mL}}{\rm{s}}} \right)}} = \frac{{F_{\rm{G}}\left( {\frac{\rm{mg}}{\rm{L}}} \right) \ast 2(L)}}{{20\left( {\frac{\rm{mg}}{\rm{mL}}} \right) \ast 3\left( {\frac{\rm{mL}}{\rm{s}}} \right)}}\\ T_{\rm{pump}},_{\rm{N}}\!:{\rm{Nitrate}}\;{\rm{pump}}\;{\rm{running}}\;{\rm{time}}\;{\rm{every}}\;8\;{\rm{h}}\;{\rm{(s)}}\\ T_{\rm{pump}},_{\rm{G}}\!:{\rm{Glucose}}\;{\rm{pump}}\;{\rm{running}}\;{\rm{time}}\;{\rm{every}}\;8\;{\rm{h}}\;{\rm{(s)}}\end{array}$$

#### Closed-loop system

Initial biomass levels (*x*_0_), glucose levels (*G*_0_) and nitrate levels (*N*_0_) were measured and used as inputs into the closed-loop system.

$${{{\mathrm{Initial}}}}\;{{{\mathrm{conditions}}}}\!\!:x_{\rm{initial}} = x_0G_{\rm{initial}} = G_0N_{\rm{initial}} = N_0$$

During the experiment, biomass levels (*x*_m_), glucose levels (*G*_m_) and nitrate levels (*N*_m_) were msured and used as inputs into the closed-loop system.

#### Light cycles

For the light cycle, two equations were built to describe and predict biomass accumulation rate and nitrate consumption rate. Unlike the open loop system, the light shielding effect was considered and the growth rate would decrease as the biomass concentration increased as described in the equation below and shown in Fig. 3c. The GSM was used to predict nutrient exchange rate (*r*_N_) based on the measured growth rate. Nitrate demand on a per liter basis (*F*_N_ (mg/L)) was fed to the bioreactor every 8 h during the light cycle.

$$\begin{array}{l}\frac{{dx}}{{dt}} = \mu x\;\mu = 0.0338 \ast \exp \left( { - 0.00174 \ast x} \right)\left( {\rm{h}^{ - 1}} \right)\\ \frac{{dN}}{{dt}} = r_{\rm{N}}x\;r_{\rm{N}}\left( {\frac{\rm{mmol}}{{gDw \ast {\rm{h}}}}} \right){\rm{was}}\;{\rm{determined}}\;{\rm{by}}\;\mu \;{\rm{and}}\;{\rm{genome}} - {\rm{scale}}\;{\rm{model}}\end{array}$$

$$\begin{array}{l}F_N = Mw_{nitrate}{\displaystyle{\int}}_t^{t + 8} {\frac{{dN}}{{dt}}dt} \\ F_N\!\!:Nitrate\;demand\;every\;8\;hours(mg/L)\end{array}$$

#### Dark cycles

For the dark cycles, three model equations were built to predict biomass accumulation rate, nitrate consumption rate and glucose consumption rate as listed below and shown in Fig. 3b. In the biomass equation, we assumed a fraction of heterotrophic biomass, *a*, was derived from autotrophic metabolism and the simulated growth rate was μ_A_. Meanwhile, some biomass was derived through heterotrophic metabolism with the simulated growth rate, *μ*_H._ The nutrient exchange rates (*r*_NA_, *r*_NH_, *r*_GH_) were determined by inputting simulated growth rates into the autotrophic and heterotrophic GSMs respectively.

$$\frac{{dx}}{{dt}} = a \ast \mu _{\rm{A}}x + \mu _{\rm{H}}x$$

$$\frac{{dN}}{{dt}} = a \ast r_{{\rm{NA}}}x + r_{{\rm{NH}}}x$$

$$\frac{{dG}}{{dt}} = r_{GH}x$$

$$\mu _{\rm{model}} = a \ast \mu _{\rm{A}} + \mu _{\rm{H}}$$

where *μ*_A_ is simulation growth rate from autotrophic metabolism, *μ*_H_ is the growth rate from heterotrophic metabolism, *r*_NA_ is nitrate exchange rate from autotrophic metabolism, *r*_NH_ is the nitrate exchange rate from heterotrophic metabolism, *r*_GH_ is the glucose exchange rate from heterotrophic metabolism.

The bolus glucose demand and nitrate demand on a per liter basis (*F*_N, model_, *F*_G,model_ (mg/L)) can be determined based on the following equations:

$$\begin{array}{l}F_{\rm{N}},{\rm{model}} = Mw_{\rm{nitrate}}{\displaystyle{\int}}_t^{t + 8} {\frac{{dN}}{{dt}}dt = Mw_{\rm{nitrate}}{\displaystyle{\int}}_t^{t + 8} {(a \ast r_{\rm{NA}}x + r_{\rm{NH}}x)dt = a \ast F_{\rm{NA}} + F_{\rm{NH}}} } \\ F_{\rm{G}},{\rm{model}} = Mw_{\rm{glucose}}{\displaystyle{\int}}_t^{t + 8} {\frac{{dG}}{{dt}}dt} \end{array}$$

$$\begin{array}{l}F_{\rm{N}},{\rm{model}}\!:{\rm{Nitrate}}\;{\rm{demand}}\;{\rm{every}}\;8\;{\rm{h}}\;{\rm{based}}\;{\rm{on}}\;{\rm{model}}\;{\rm{simulation}}\;({\rm{mg/L}})\\ F_{\rm{G}},{\rm{model}}\!:{\rm{Glucose}}\;{\rm{demand}}\;{\rm{every}}\;8\;{\rm{h}}\;{\rm{based}}\;{\rm{on}}\;{\rm{model}}\;{\rm{simulation}}({\rm{mg/L}})\end{array}$$

The calculated growth rate (*μ*_c_) and calculated nutrient demand (*F*_NC_ and *F*_GC_) were determined from feedback measurements (*X*_*m*_, *G*_*m*_, *N*_*m*_) at *t* and *t* − 8 using the following equations:

$$\begin{array}{l}\mu _{\rm{c}} = \frac{{{{{\mathrm{ln}}}}\left( {x_{m,t}/x_{m,t - 8}} \right)}}{{t - (t - 8)}}\\ F_{{\rm{NC}}} = N_{m,t} - N_{m,t - 8}\\ F_{{\rm{GC}}} = G_{m,t} - G_{m,t - 8}\end{array}$$

Next, we applied a fitting objective function (*J*) to minimize the difference between calculated values and simulated (model) values in order to estimate the optimal parameter values (*a*, *μ*_A_, *μ*_H_, *r*_NA_, *r*_NH_, *r*_GH_) for dictating the actual nitrate and glucose feeds to the bioreactor.

$$J = \left( {\frac{{\mu _{\rm{model}} - \mu _{\rm{c}}}}{{\mu _{\rm{c}}}}} \right)^2 + \left( {\frac{{F_{\rm{G,model}} - F_{\rm{GC}}}}{{F_{\rm{GC}}}}} \right)^2 + \left( {\frac{{F_{\rm{N,model}} - F_{\rm{NC}}}}{{F_{\rm{NC}}}}} \right)^2$$

The actual bolus nitrate demand (*F*_N_) and the glucose demand (*F*_G_) were thus determined by using values obtained from this fitting objective function. Based on these estimations, two separate pumps would pump glucose and nitrate every 8 h at the beginning of the dark cycle period.

The peristaltic pumps will pump nitrate and glucose into the bioreactor (*T*_pump,N_, *T*_pump,G_) based on nitrate and glucose demand on a per liter basis (*F*_N_, *F*_G_ (mg/L)), feed concentration (*C*_N_, *C*_G_ (mg/mL)), volume of the bioreactor (*V* (*L*)) and pump volumetric flow rate speed (*Q* (mL/s)).

$$\begin{array}{l}T_{\rm{pump}},_{\rm{N}} = \frac{{F_{\rm{N}}\left( {\frac{\rm{mg}}{{\rm{L}}}} \right) \ast V(L)}}{{C_{\rm{N}}\left( {\frac{\rm{mg}}{\rm{mL}}} \right) \ast Q\left( {\frac{\rm{mL}}{\rm{s}}} \right)}} = \frac{{F_{\rm{N}}\left( {\frac{\rm{mg}}{{\rm{L}}}} \right) \ast 2(L)}}{{5\left( {\frac{\rm{mg}}{\rm{mL}}} \right) \ast 3\left( {\frac{\rm{mL}}{\rm{s}}} \right)}}\\ T_{\rm{pump}},_{\rm{G}} = \frac{{F_{\rm{G}}\left( {\frac{\rm{mg}}{{\rm{L}}}} \right) \ast V(L)}}{{C_{\rm{G}}\left( {\frac{\rm{mg}}{\rm{mL}}} \right) \ast Q\left( {\frac{\rm{mL}}{\rm{s}}} \right)}} = \frac{{F_{\rm{G}}\left( {\frac{\rm{mg}}{{\rm{L}}}} \right) \ast 2(L)}}{{20\left( {\frac{\rm{mg}}{\rm{mL}}} \right) \ast 3\left( {\frac{\rm{mL}}{\rm{s}}} \right)}}\end{array}$$

$$\begin{array}{l}T_{\rm{pump}},_{\rm{N}}\!:{\rm{Nitrate}}\;{\rm{pump}}\;{\rm{running}}\;{\rm{time}}\;{\rm{every}}\;8\;{\rm{h}}\;({\rm{s}})\\ T_{\rm{pump}},_{\rm{G}}\!:{\rm{Glucose}}\;{\rm{pump}}\;{\rm{running}}\;{\rm{time}}\;{\rm{every}}\;8\;{\rm{h}}\;({\rm{s}})\end{array}$$

## Supplementary information

Supplementary Information

## Data Availability

The data that support the findings of this study are available from the corresponding author upon reasonable request.

## Code availability

The codes that support the findings of this study are provided in supplementary information.
