# Supplementary material for: Optimization of nutrient utilization efficiency and productivity for algal cultures under light and dark cycles using genome-scale model process control
Source: NPJ Syst Biol Appl. 2023 Mar 15;9:7. doi: 10.1038/s41540-022-00260-7 (PMC10017758; doi:10.1038/s41540-022-00260-7)
Supplement: Supplementary file 1 — Supplementary Information [file 41540_2022_260_MOESM1_ESM.pdf]

Supplementary material

Model	Condition	Biomass composition
iCZ946-PAT1	Autotrophic<br>Nitrogen replete	49% Amino acid, 16% Lipid, 25% Carbohydrate
iCZ946-PAT2	Autotrophic<br>Nitrogen depleted	45% Amino acid, 10% Lipid, 35% Carbohydrate
iCZ946-PAT3	Autotrophic<br>Nitrogen depleted	22% Amino acid, 25% Lipid, 43% Carbohydrate
iCZ946-PAT4	Autotrophic<br>Nitrogen depleted	20% Amino acid, 29% Lipid, 43% Carbohydrate
iCZ946-PAT5	Autotrophic<br>Nitrogen depleted	16% Amino acid, 50% Lipid, 30% Carbohydrate
iCZ946-PAT6	Autotrophic<br>Nitrogen depleted	15% Amino acid, 55% Lipid, 27% Carbohydrate
iCZ946-HT1	Heterotrophic<br>Nitrogen replete	35% Amino acid, 27% Lipid, 33% Carbohydrate
iCZ946-HT2	Heterotrophic<br>Nitrogen depleted	32% Amino acid, 27% Lipid, 35% Carbohydrate
iCZ946-HT3	Heterotrophic<br>Nitrogen deplete	28% Amino acid, 27% Lipid, 37% Carbohydrate
iCZ946-HT4	Heterotrophic<br>Nitrogen depleted	21% Amino acid, 27% Lipid, 34% Carbohydrate
iCZ946-HT5	Heterotrophic<br>Nitrogen depleted	18% Amino acid, 27% Lipid, 34% Carbohydrate

Supplementary Table. 1 Models used in this study. All the models were obtained from Zuniga et al 2018.

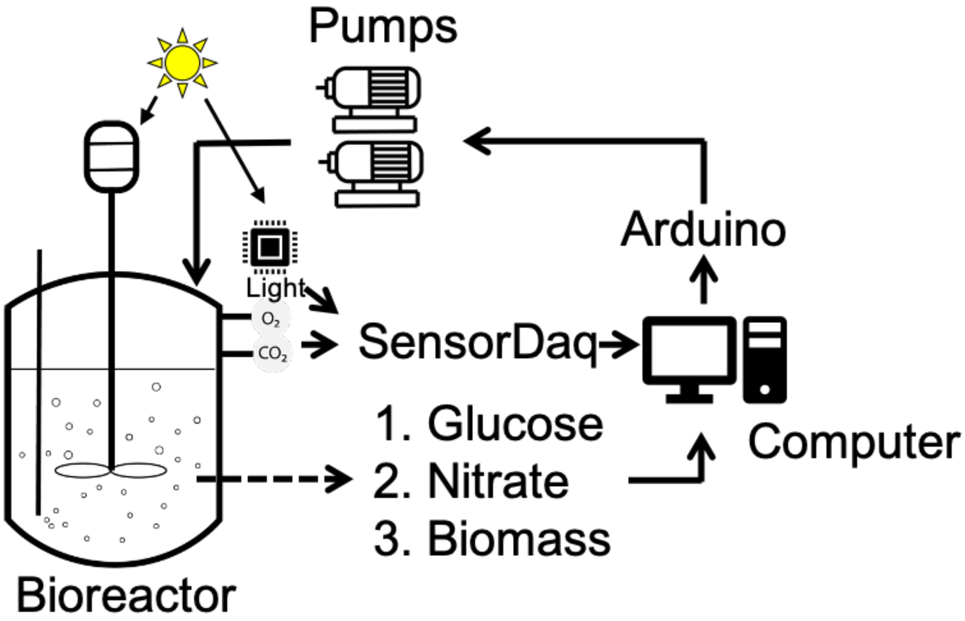

Supplementary Figure. 1 Experimental setup for the control system.

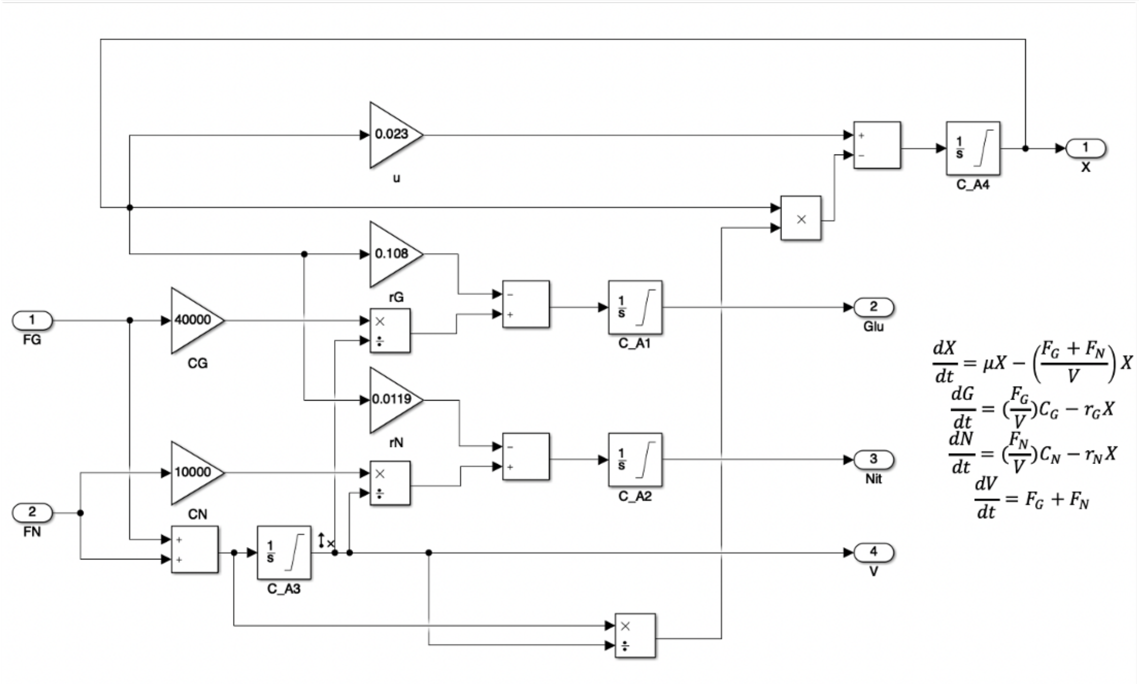

Supplementary Figure. 2 Kinetic model inside the bioreactor block (blue) in **Fig. 5a**.

Matlab Code

- *Open_loop.m* runs the code for open-loop experiment.
- *Closed_loop.m* runs the code for closed-loop experiment.
- *optimizemodelV3.m* calculates the optimal nitrate supply for autotrophic conditions in closed-loop experiment.
- *parameterV2.m* calculates optimal parameters for heterotrophic conditions in closed-loop experiment.

Open_loop.m

```
% This is the code for the open-loop experiment

mi = 42.52;

global probeChip
nitPumpPin = 'D9';%pin for nitrate pump
gluPumpPin = 'D8';%pin for glucose pump

% on and off vary depending on the relay being used to control pumps
on = 0;
off = 1;

% Set up timer =0 for nutrient supplies
total_nita = 0;
total_nith = 0;
total_glua = 0;
total_gluh = 0;
total_nit = 0;
total_glu = 0;

nitpertimea = [];
nitpertimeh = [];
glupertimea = [];
glupertimeh = [];
nitpertime = [];
glupertime = [];

% Set up probes
o = sdaq.createSession;
sdaq.addSensor(o,1,sdaq.Sensors.LightSensor150000lux);
sdaq.addSensor(o,2,sdaq.Sensors.CO2Gas);
sdaq.addSensor(o,3,sdaq.Sensors.OxygenGas);

update =[];
newdata =[];
pause('on');
count = 0;
biomass = 0;
c = [0 0 0 0 0 0];
V = 2;

% Collect data for 5 days
while floor(c(3)) ~= 15
    tic
    c = clock;

    if floor(c(5)) == 30 && floor(c(6)) == 0 || floor(c(5)) == 0 &&
floor(c(6)) == 0

        % Run data collection function. This function worked every 1 h.
        rawdata = o.inputSingleScan();

        % Change units
```

```
        rawdata(:,1)= rawdata(:,1)*38424; %(lux)
        rawdata(:,2)= rawdata(:,2)*1.9809 + 0.1844; %(%CO2)
        rawdata(:,3)= rawdata(:,3)*25.959 - 36.942; %(%O2)

        % Nitrogen gas ratio didn't change
        x=(1-0.21-0.05)/(1-rawdata(:,2)/100-rawdata(:,3)/100);

        % Calculate CTR(mmol/L/h) capture how much CO2 420 ml/min
        rawdata(:,4)= 0.42/3/24.5*(0.05-rawdata(:,2)/100*x)*1000*60;

        % Calculate OTR(mmol/L/h) produce how much O2 420 ml/min
        rawdata(:,5)= 0.42/3/24.5*(rawdata(:,3)/100*x-0.21)*1000*60;

        % Calculate RQ
        rawdata(:,6)= rawdata(:,4)./rawdata(:,5);

        time_d = count;
        newdata = [time_d, rawdata];
        update = [update; newdata];

        subplot(2,2,1);

            yyaxis right
            plot(update(:,1),update(:,2))
            ylim([0 12000])
            hold on
            ylabel('Light intensity (lux)');

            yyaxis left
            plot(update(:,1),update(:,3))
            hold on
            plot(update(:,1),update(:,4))
            hold off

            xlabel('Time (hr)');
            ylabel({'CO2 level(%),O2 level(%)'});
            legend('light','CO2 level','O2 level','location','northwest');

            drawnow
            subplot(2,2,2);

            plot(update(:,1),update(:,5),'color','r') %CTR
            xlabel('Time (hr)');
            ylabel('CTR(mmol/L/h),OTR(mmol/L/h)');
            ylim([-5 250])
            hold on
            plot(update(:,1),update(:,6),'color','b') %OTR
            legend({'CTR','OTR'},'location','northwest');
            drawnow

        % Start pumping nutrients. This function only worked for 2am, 10am
        % and 6pm
        if 4 == c(4) && 0 == c(5) && 0 == floor(c(6)) || 12 == c(4) && 0 ==
c(5) && 0 == floor(c(6)) || 20 == c(4) && 0 == c(5) && 0 == floor(c(6))
            tf = 8;
            t0 = 0;
```

```
            syms t
            if c(4) == 12
                if count == 0
                    z = mi*exp(0.023*t); %mg/L*h
                    biomass = mi * exp(0.023 * 8)
                    inter = double(int(z,t,t0,tf));
                else
                    z = biomass*exp(0.023*t); %mg/L*h
                    biomass = biomass * exp(0.023 * 8)
                    inter = double(int(z,t,t0,tf));
                end
            elseif c(4) == 4 || c(4) == 20
                z = biomass*exp(0.035*t); %mg/L*h
                biomass = biomass * exp(0.035 * 8)
                inter = double(int(z,t,t0,tf));
            end
            if 4 == c(4) && 0 == c(5) && 0 == floor(c(6)) || 20 == c(4) &&  0
== c(5) && 0 == floor(c(6))
                disp('light')
                t_auto_nit= 0.1823*85e-3*inter*V/(3*5); %Calculate how many
secs did nitrate pump work
                V = V + 0.003*t_auto_nit
                writeDigitalPin(probeChip, nitPumpPin, on)
                pause(t_auto_nit);% Pause is in seconds
                writeDigitalPin(probeChip, nitPumpPin, off)

                total_nita = total_nita + t_auto_nit*5*3/V; % mg/L
                nitpertimea=[nitpertimea;count,total_nita]; % mg/L
                %time = [time;count,light];

                total_glua = total_glua +0; % mg/L
                glupertimea = [glupertimea; count,total_glua];

            elseif 12 == c(4) && 0 == c(5) && 0 == floor(c(6))

                disp('dark')
                t_hetero_nit= 0.1417*85e-3*inter *V/(3*5); %Calculate how
many secs did nitrate pump work
                t_hetero_glu= 0.6426*180e-3*inter*V/(3*20);
%Calculate how many secs did nitrate pump work

                V = V + 0.003*(t_hetero_nit+t_hetero_glu)

                writeDigitalPin(probeChip, nitPumpPin, on)
                pause(t_hetero_nit);% Pause is in seconds
                writeDigitalPin(probeChip, nitPumpPin, off)

                writeDigitalPin(probeChip, gluPumpPin, on)
                pause(t_hetero_glu);% Pause is in seconds
                writeDigitalPin(probeChip, gluPumpPin, off)

                total_nith = total_nith + t_hetero_nit *3*5/V; % mg/L
                nitpertimeh=[nitpertimeh;count,total_nith]; % mg/L

                total_gluh = total_gluh + t_hetero_glu *3*20/V; % mg/L
                glupertimeh = [glupertimeh; count,total_gluh];
```

```
                %time = [time;count,dark];
            end

        end
        total_nit = total_nita + total_nith;
        nitpertime = [nitpertime;count,total_nit];
        total_glu = total_glua + total_gluh;
        glupertime = [glupertime;count,total_glu];

        subplot(2,2,3);
        yyaxis right
        plot(nitpertime(:,1),nitpertime(:,2))
        ylim([0 200])
        ylabel('Nitrate(mg/L)');
        hold on

        yyaxis left
        plot(glupertime(:,1),glupertime(:,2))
        ylim([0 500])
        ylabel('Glucose(mg/L)');
        hold off

        xlabel('Time (min)')
        ylabel({'Glucose (mg/L)'})
        legend({'Nitrate Fed','Glucose Fed'},'location','northwest')

        drawnow

        totaldata = [rawdata,total_nit,total_glu]

        count = count+1
    end
    h = toc;
    pause(1-h)
end
```

Closed_loop.m

```
% Closed-loop system

%--------------------------------------------------------------------------
% In this system, we considered light blocking effect on cell growth under
light cycle.
% We also considered autotrophic growth under dark cycles.
%--------------------------------------------------------------------------

% on and off vary depending on the relay being used to control pumps
on =
0;
off = 1;

% Set up timer =0 for nutrient supplies
total_nita = 0;
total_nith = 0;
total_glua = 0;
total_gluh = 0;
 total_nit = 0;
 total_glu = 0;

nitpertimea = [];
nitpertimeh = [];
glupertimea = [];
glupertimeh = [];
 nitpertime = [];
 glupertime = [];

% Set up probes
o = sdaq.createSession;
sdaq.addSensor(o,1,sdaq.Sensors.LightSensor150000lux);
sdaq.addSensor(o,2,sdaq.Sensors.CO2Gas);
sdaq.addSensor(o,3,sdaq.Sensors.OxygenGas);

update =[];
newdata =[];
pause('on');
count = 0;
biomass = 0;
c = [0 0 0 0 0 0];
V = 2;
global y

% Collect data for 5 days
while floor(c(3)) ~= 27

    tic
    c = clock;

    if floor(c(5)) == 0 && floor(c(6)) == 0 || floor(c(5)) == 30 &&
floor(c(6)) == 0

        % This function worked to collect data every 30 min.
        rawdata = o.inputSingleScan();
```

```
% Change units
rawdata(:,1) = rawdata(:,1)*38424; %(lux)
rawdata(:,2) = rawdata(:,2)*1.9809 + 0.1844; %(%CO2)
rawdata(:,3) = rawdata(:,3)*25.959 - 36.942; %(%O2)

% Nitrogen gas ratio didn't change
q = (1-0.21-0.05)/(1-rawdata(:,2)/100-rawdata(:,3)/100);

% Calculate CTR(mmol/L/h) capture how much CO2 420 ml/min
rawdata(:,4) = 0.42/3/24.5*(0.05-rawdata(:,2)/100*q)*1000*60;

% Calculate OTR(mmol/L/h) produce how much O2 420 ml/min
rawdata(:,5) = 0.42/3/24.5*(rawdata(:,3)/100*q-0.21)*1000*60;

% Calculate RQ
rawdata(:,6) = rawdata(:,4)./rawdata(:,5);

 time_d = count;
newdata = [time_d, rawdata];
 update = [update; newdata];

subplot(2,2,1);

    yyaxis right
    plot(update(:,1),update(:,2))
    ylim([0 12000])
    hold on
    ylabel('Light intensity (lux)');

    yyaxis left
    plot(update(:,1),update(:,3))
    hold on
    plot(update(:,1),update(:,4))
    hold off

    xlabel('Time (h)');
    ylabel({'CO2 level(%),O2 level(%)'});
    legend('light','CO2 level','O2 level','location','northwest');

    drawnow
    subplot(2,2,2);

    plot(update(:,1),update(:,5),'color','r') %CTR
    xlabel('Time (h)');
    ylabel('CTR (mmol/L/h),OTR (mmol/L/h)');
    ylim([-5 250])
    hold on
    plot(update(:,1),update(:,6),'color','b') %OTR
    legend({'CTR','OTR'},'location','northwest');
    drawnow

% The function worked every 8 h to pump nutrients.
```

```
        if 12 == c(4) && 0 == c(5) && 0 == floor(c(6)) || 20 == c(4) && 0 ==
c(5) && 0 == floor(c(6)) || 4 == c(4) && 0 == c(5) && 0 == floor(c(6))
            t0 = 0;
            tf = 8;
            syms t
            if 12 == c(4) && 0 == c(5)
                if count == 0

                    disp ('dark cycle and all the setting')
                    rg_hh1p = -0.6426
                    rn_hh1p = -0.1417
                     u_hh1p = 0.023

                        x1p = 0
                    rn_ha1p = -0.1823
                     u_ha1p = 0.035

                else

                    disp('dark cycle and all the setting')
                    y = xlsread ('realtime.xlsx'); % Real-time data
                    b_t0(1,1) = y(1,3);
                    b_t0(1,2) = y(1,6);
                    b_t0(1,3) = y(1,9);
                    biomass = (b_t0(1,1)+b_t0(1,2)+b_t0(1,3))/3

                    [x1e,u_hh1e,u_ha1e,rg_hh1e,rn_hh1e,rn_ha1e]=parameterV2()

                    z_a = biomass*x1e*exp(u_a*t);
                    inter_a = double(int(z_a,t,t0,tf)); %mg/L*h
                    z_h = biomass*exp(u_h*t);
                    inter_h = double(int(z_h,t,t0,tf)); %mg/L*h

                    FG_opt = inter_h*rg_hh1e*180e-3;
                    FN_opt = (inter_a*rn_ha1e+inter_h*rn_hh1e)*85e-3;
                end
            elseif 20 == c(4) && 0 == c(5)

                    y = xlsread ('realtime.xlsx'); % Real-time data
                    b_t0(1,1) = y(1,3);
                    b_t0(1,2) = y(1,6);
                    b_t0(1,3) = y(1,9);
                    biomass = (b_t0(1,1)+b_t0(1,2)+b_t0(1,3))/3

                    tspan = [0 8];
                    z0 = biomass;
                    [t,z] = ode45(@(t,z) 0.0338*exp(-0.00174*z)*z, tspan,
z0);
                    v = cumtrapz(t,z);
                    inter = v(end);

                    u = log(z(end)/biomass)/8;
                    rn = optimizemodelV3(1,u)

                    biomass_4am = z(end);
                    FN_opt = (inter*rn)*85e-3;
```

```
            elseif 4 == c(4) && 0 == c(5)

                    tspan = [0 8];
                    z0 = biomass_4am;
                    [t,z] = ode45(@(t,z) 0.0338*exp(-0.00174*z)*z, tspan,
z0);
                    v = cumtrapz(t,z);
                    inter = v(end);

                    u = log(z(end)/biomass_4am)/8;
                    rn = optimizemodelV3(1,u)

                    FN_opt = (inter*rn)*85e-3;

            end

            global probeChip
            gluPumpPin = 'D8'; %pin for glucose pump
            nitPumpPin = 'D9'; %pin for nitrate pump

            if 20 == c(4) && 0 == c(5) && 0 == floor(c(6)) || 4 == c(4) &&  0
== c(5) && 0 == floor(c(6))

                disp('light')
                t_auto_nit= -FN_opt*V/(3*10); %Calculate how many secs did
nitrate pump work
                V = V + 0.003*t_auto_nit

                writeDigitalPin(probeChip, nitPumpPin, on)
                pause(t_auto_nit);% Pause is in seconds
                writeDigitalPin(probeChip, nitPumpPin, off)

                total_nita = total_nita + t_auto_nit*3*10/V; % mg/L
                nitpertimea=[nitpertimea;count,total_nita]; % mg/L
                %time = [time;count,light];

                total_glua = total_glua +0; % mg/L
                glupertimea = [glupertimea; count,total_glua];

            elseif 12 == c(4) && 0 == c(5) && 0 == floor(c(6))

                disp('dark')
                t_hetero_nit= -FN_opt*V/(3*10); %Calculate how many secs did
nitrate pump work
                t_hetero_glu= -FG_opt*V/(3*40); %Calculate how many secs did
glucose pump work

                V = V + 0.003*(t_hetero_nit+t_hetero_glu)

                writeDigitalPin(probeChip, nitPumpPin, on)
                pause(t_hetero_nit);% Pause is in seconds
                writeDigitalPin(probeChip, nitPumpPin, off)

                writeDigitalPin(probeChip, gluPumpPin, on)
```

```
                pause(t_hetero_glu);% Pause is in seconds
                writeDigitalPin(probeChip, gluPumpPin, off)

                total_nith = total_nith + t_hetero_nit*3*10/V; % mg/L
                nitpertimeh=[nitpertimeh;count,total_nith];    % mg/L

                total_gluh = total_gluh + t_hetero_glu*3*40/V; % mg/L
                glupertimeh = [glupertimeh; count,total_gluh]; % mg/L
                %time = [time;count,dark];

            end

        end
        total_nit = total_nita + total_nith;
        nitpertime = [nitpertime;count,total_nit];
        total_glu = total_glua + total_gluh;
        glupertime = [glupertime;count,total_glu];

        subplot(2,2,3);
        yyaxis right
        plot(nitpertime(:,1),nitpertime(:,2))
        ylim([0 200])
        ylabel('Nitrate (mg/L)');
        hold on

        yyaxis left
        plot(glupertime(:,1),glupertime(:,2))
        ylim([0 500])
        ylabel('Glucose (mg/L)');
        hold off

        xlabel('Time (h)')
        ylabel({'Glucose (mg/L)'})
        legend({'Nitrate Fed','Glucose Fed'},'location','northwest')

        drawnow

        totaldata = [rawdata,total_nit,total_glu]

        count = count+1
    end
    h = toc;
    pause(1-h)
end
```

optimizemodelV3.m

```
function [rN_x]=optimizemodelV3(x,u)

%---------------------------------------------------------------------------
% We took the biomass compositions from 6 different autotrophic models and
% used polynomial curves to fit the data. This script was used to find
optimal
% biomass compositions to achieve adaptive model predictive control.
%---------------------------------------------------------------------------

modelPAT1 = readCbModel('iCZPA_T1.xml');
model = modelPAT1;

% Change biomass content
p = xlsread ('biomass parameters_a.xlsx');

for i=1:1770
    model.S(i,62) = p(i,1)*x^3+ p(i,2)*x^2 + p(i,3)*x + p(i,4);
end

% Nitrate uptake rate from the model
model = changeBounds(model, 'Biomass_Cvu_auto-', u,'b');
model = changeBounds(model, 'EX_no3(e)', 0,'u');
model = changeBounds(model, 'EX_no3(e)', -1000,'l');
model = defineObj(model, 'EX_no3(e)','max');
sol_nit = solveFBA(model);
rN_x = sol_nit.f;

end
```

parameterV2.m

```
function [x1e,u_hh1e,u_ha1e,rg_hh1e,rn_hh1e,rn_ha1e]=parameterV2()

%-----------------------------------------------------------------------
% This script was built to find optimal parameters by using experimental
% data.
%-----------------------------------------------------------------------

% Input all the data: biomass, glucose, nitrate and model prediction
parameters.
global x
x = xlsread ('updateV2.xlsx');
t = zeros(4);
biomass = zeros(4,3);
nitrate = zeros(4,3);
glucose = zeros(4,3);
th = zeros(3);
for i= 1:4
t(i) = x(i,1);
biomass(i,1) = x(i,4);
biomass(i,2) = x(i,7);
biomass(i,3) = x(i,10);
nitrate(i,1) = x(i,11);
nitrate(i,2) = x(i,12);
nitrate(i,3) = x(i,13);
glucose(i,1) = x(i,14);
glucose(i,2) = x(i,15);
glucose(i,3) = x(i,16);
end
for i = 1:3
th(i) = t(i+1)- t(i);
end
% rn_"hh1p" means "heterotropic culture" "heterotrophic growth1" "prediction"
x1p = x(1,17);
rn_hh1p = x(1,18);
rg_hh1p = x(1,19);
u_hh1p = x(1,20);
rn_ha1p = x(2,19);
u_ha1p = x(2,20);

%-----------------------------------------------------------------------
% Based on experimental data, calculate some parameters for optimization
u_hall1e = zeros(1,3);
FG_hall1e = zeros(1,3);
FN_hall1e = zeros(1,3);
u_a1e = zeros(1,3);
FG_a1e = zeros(1,3);
FN_a1e = zeros(1,3);
for i= 1:3
u_hall1e(1,i) = log(biomass(2,i)/biomass(1,i))/th(1);
FG_hall1e(1,i) = glucose(2,i)-glucose(1,i);
FN_hall1e(1,i) = nitrate(2,i)-nitrate(1,i);
u_a1e(1,i) = log(biomass(4,i)/biomass(3,i))/th(3);
FG_a1e(1,i) = glucose(4,i)-glucose(3,i);
```

```
        FN_a1e(1,i) = nitrate(4,i)-nitrate(3,i);
    end

%---------------------------------------------------------------------------
% Based on the data obtained from previous heterotrophic cycles, calculate
% x, ua, ug, FG, FN (0-8h)
    options = optimoptions(@fmincon,'Display','iter','MaxIter',3);
    lb = [0,0,0];
    ub = [1,0.03,0.04];
    A = [];
    b = [];
    Aeq = [];
    beq = [];
    x0 = [x1p,u_hh1p,u_ha1p];
    x = fmincon (@helper_func,x0,A,b,Aeq,beq,lb,ub,[],options)

    function F = helper_func(x)
    [f,g,h] = findratio(x);
    F = ((f-mean(u_hall1e))/mean(u_hall1e))^2+((-g-
mean(FG_hall1e))/mean(FG_hall1e))^2+((-h-mean(FN_hall1e))/mean(FN_hall1e))^2
    end

% % "e" stands for "experiment"
      x1e = x(1)
    u_hh1e = x(2)
    u_ha1e = x(3)

    modelHT1 = readCbModel('iCZH_T1.xml');
    modelPAT1 = readCbModel('iCZPA_T1.xml');

    modelHT1 = changeBounds(modelHT1, 'Biomass_Cvu_hetero-', u_hh1e,'b');
    modelHT1 = changeBounds(modelHT1, 'EX_glc-A(e)', 0,'u');
    modelHT1 = changeBounds(modelHT1, 'EX_glc-A(e)', -1000,'l');
    modelHT1 = defineObj(modelHT1, 'EX_glc-A(e)','max');
    sol_glu = solveFBA(modelHT1, 'max');
    rg_hh1e = sol_glu.f;

    modelHT1 = changeBounds(modelHT1, 'EX_glc-A(e)', rg_hh1e,'b');
    modelHT1 = changeBounds(modelHT1, 'EX_no3(e)', 0,'u');
    modelHT1 = changeBounds(modelHT1, 'EX_no3(e)', -1000,'l');
    modelHT1 = defineObj(modelHT1, 'EX_no3(e)','max');
    sol_nit = solveFBA(modelHT1, 'max');
    rn_hh1e = sol_nit.f;

    modelPAT1 = changeBounds(modelPAT1, 'Biomass_Cvu_auto-', u_ha1e,'b');
    modelPAT1 = changeBounds(modelPAT1, 'EX_no3(e)', 0,'u');
    modelPAT1 = changeBounds(modelPAT1, 'EX_no3(e)', -1000,'l');
    modelPAT1 = defineObj(modelPAT1, 'EX_no3(e)','max');
    sol_nit = solveFBA(modelPAT1, 'max');
    rn_ha1e = sol_nit.f;

end
```
